# Secretion of Mast Cell Inflammatory Mediators Is Enhanced by CADM1-Dependent Adhesion to Sensory Neurons

**DOI:** 10.3389/fncel.2019.00262

**Published:** 2019-06-18

**Authors:** Rania Magadmi, Judit Meszaros, Zoheir A. Damanhouri, Elizabeth P. Seward

**Affiliations:** ^1^Department of Biomedical Science, University of Sheffield, Sheffield, United Kingdom; ^2^Department of Pharmacology, Faculty of Medicine, King Abdulaziz University, Jeddah, Saudi Arabia

**Keywords:** mast cells, pain, allergy, IGE receptor, CADM1, synCAM1, sensory neurons

## Abstract

Neuroimmune interactions are important in the pathophysiology of many chronic inflammatory diseases, particularly those associated with alterations in sensory processing and pain. Mast cells and sensory neuron nerve endings are found in areas of the body exposed to the external environment, both are specialized to sense potential damage by injury or pathogens and signal to the immune system and nervous system, respectively, to elicit protective responses. Cell adhesion molecule 1 (CADM1), also known as SynCAM1, has previously been identified as an adhesion molecule which may couple mast cells to sensory neurons however, whether this molecule exerts a functional as well as structural role in neuroimmune cross-talk is unknown. Here we show, using a newly developed *in vitro* co-culture system consisting of murine bone marrow derived mast cells (BMMC) and adult sensory neurons isolated from dorsal root ganglions (DRG), that CADM1 is expressed in mast cells and adult sensory neurons and mediates strong adhesion between the two cell types. Non-neuronal cells in the DRG cultures did not express CADM1, and mast cells did not adhere to them. The interaction of BMMCs with sensory neurons was found to induce mast cell degranulation and IL-6 secretion and to enhance responses to antigen stimulation and activation of FcεRI receptors. Secretion of TNFα in contrast was not affected, nor was secretion evoked by compound 48/80. Co-cultures of BMMCs with HEK 293 cells, which also express CADM1, while also leading to adhesion did not replicate the effects of sensory neurons on mast cells, indicative of a neuron-specific interaction. Application of a CADM1 blocking peptide or knockdown of CADM1 in BMMCs significantly decreased BMMC attachment to sensory neurites and abolished the enhanced secretory responses of mast cells. In conclusion, CADM1 is necessary and sufficient to drive mast cell-sensory neuron adhesion and promote the development of a microenvironment in which neurons enhance mast cell responsiveness to antigen, this interaction could explain why the incidence of painful neuroinflammatory disorders such as irritable bowel syndrome (IBS) are increased in atopic patients.

## Introduction

Mast cells are best known for their role in allergic diseases. The symptoms of allergic reactions are instigated by the secretion of a plethora of pro-inflammatory mediators from mast cells following antigen-dependent crosslinking of IgE receptors (FcεRI) (Galli and Tsai, 2012). These mediators include preformed molecules stored in granules such as histamine, serotonin, adenosine 5′-triphosphate (ATP), proteases, tumor necrosis factor-α (TNFα), chemokines, and peptides, as well as de novo synthesized cytokines, growth factors and lipid mediators (Sismanopoulos et al., 2012). Since mast cells are tissue resident cells, the mediators they secrete influence the function of nearby cells expressing cognate receptors. Conversely, mast cells also express a wide variety of other types of receptors whose activation by local mediators may intern amplify antigen-induced responses (Gilfillan et al., 2009), while much is known about mast cell cross-talk with other innate and adaptive immune cells, more recently, their contribution to neuroimmune signaling is also increasingly being recognized (Undem and Taylor-Clark, 2014; Voisin et al., 2017).

Evidence is accumulating that mast cells may contribute to pain experienced in conditions whose pathology involves tissues lying at the interface of the external environment such as intestines, bladder, uterus, airways, skin and meninges (Aich et al., 2015; Gupta and Harvima, 2018). Indeed, mast cells have shown preference to attach to substance P (subP)and calcitonin gene related peptide (CGRP)-positive sensory neurons in human and rat intestine (Stead et al., 1987), respiratory tract (Alving et al., 1991) dura matter (Rozniecki et al., 1999), and other tissues (Spanos et al., 1997; Pavlovic et al., 2008). The number of contacts between mast cells and neurons is increased during infection (Stead et al., 1987), allergic conditions (El-Nour et al., 2005), and inflammatory conditions such as irritable bowel syndrome (Barbara et al., 2004; Theoharides and Conti, 2004) and this correlates to pain (Barbara et al., 2007; Ohman and Simrén, 2010; Di Nardo et al., 2014). Mast cell granule-derived mediators and cytokines, including IL-6 and TNF-α, in turn have been shown to sensitize nociceptors (von Banchet et al., 2005; Barbara et al., 2007; Hensellek et al., 2007) and contribute directly to neurogenic inflammation and pain signaling (Aich et al., 2015; Wouters et al., 2016; Gupta and Harvima, 2018). Knowledge of the adhesion molecules regulating mast-cell sensory neuron contacts may therefore provide new insight into disease mechanisms and strategies for intervention.

Cell adhesion molecule 1 (CADM1, also known as SynCAM 1, Necl-2, SgIgSF, TSLC-1) is reported to contribute to mast cell interactions with neurons (Furuno et al., 2005; Hagiyama et al., 2011), fibroblasts and smooth muscle cells (Moiseeva et al., 2013b). CADM1 is one of four related glycoproteins with a common structure consisting of three extracellular Ig-like domains, a transmembrane region and short conserved cytoplasmic domain that binds adaptor proteins linking it to the cytoskeleton and other intracellular partners (Biederer, 2006). CADM1, 2, 3 can each form weak trans homophilic interactions, while CADM1/2 and CADM3/4 interactions produce strong heterophilic adhesions in neurons (Fogel et al., 2007). Mutations in CADM1 have been implicated in autism spectrum disorder, and its expression is increased in Rett syndrome (Nectoux et al., 2010) which is associated with altered peripheral mechanosensory transduction (Orefice et al., 2016). One study performed to date examining mast cell – sensory neuron adhesions, failed to detect CADM protein expression but did find evidence for nectin-3 mRNA expression in dorsal root ganglia. Neutralizing antibodies aimed at disrupting CADM1/nectin-3 heterophilic interactions reduced mast cell adhesion (Furuno et al., 2012; Moiseeva et al., 2013b). However, since neuronal expression of CADM1 is developmentally regulated (Fogel et al., 2007; Hagiyama et al., 2011; Moiseeva et al., 2013a,b), and the only study performed to date used neurons isolated from newborn mice, whether CADM1 contributes to functional interactions between mature sensory neurons and mast cells remains an open question. It is also unknown how adhesion between sensory neurons and mast cells modulates their responses to allergic activation. To address these questions, we established a co-culture system between adult sensory neurons isolated from dorsal root ganglia (DRG) and functionally mature mast cells generated from haematopoetic stem cells found in bone marrow derived mast cells (BMMCs). Protein expression analysis, showed that distinct variants of CADM1 are expressed in sensory neurons and mast cells. Knockdown of CADM1 in mast cells abolished their adhesion to sensory neurons, conversely blocking CADM1 on sensory neurons with a neutralizing antibody inhibited mast cell adhesion. Functional analysis of mast cells in co-culture furthermore revealed that CADM1-dependent interactions with sensory neurons induced degranulation and IL-6 synthesis, and significantly enhanced FcεRI-activated secretory responses. Separation of the sensory neurons from the mast cells by a porous membrane prevented the effects of co-culture, as did knockdown of CADM1, showing that the functional interaction was adhesion dependent. While the effects of mast cell derived mediators on sensory neurons are well documented, to our knowledge this is the first demonstration of a reciprocal adhesion-dependent effect of sensory neurons on mast cells and their responsiveness to antigen, emphasizing the important role that neuro-immune interactions contribute to allergic diseases.

## Materials and Methods

All animals were maintained on a 12-h light/dark cycle in a temperature-controlled environment and given food *ad libitum*. All animal procedures were conducted under the Animal (Scientific Procedures) Act 1986, and approved by the UK Home Office.

### Bone Marrow-Derived Mast Cells (BMMC) Cell Culture

Bone marrow-derived mast cells were isolated from 8 to 12 week-old C57BL6 wild type mice as described previously (Furuno and Nakanishi, 2011) with modifications. After the mice were sacrificed, bone marrow was collected from the tibia and the femur by repeated flushing using a 27-G needle syringe filled with calcium- and magnesium- free phosphate buffer solution (PBS, PAN Biotech, Germany). Cells were collected and centrifuged at 340 × *g* for 10 min at 4°C. The pellets obtained were re-suspended with 2-ml lysis buffer [0.83% ammonium chloride, 0.168% Na-carbonate, 1 mM EDTA (pH 7.3)], in which they were incubated for 10 min at room temperature to induce lysis of red blood cells. The lysed cells were centrifuged and resuspended with Iscove’s Modified Dulbecco’s Media (IMDM, Lonza, United Kingdom). For cell culture, complete medium was supplemented with 10% heat-inactivated fetal calf serum (FCS, Gibco, United Kingdom), 1% MEM Vitamin (Gibco, United Kingdom), 1% of sodium pyruvate (Gibco, United Kingdom), 100 IU/ml Penicillin, 100 μg/ml streptomycin (PAA Laboratories, United Kingdom), and 0.1 mM non-essential amino acid (Gibco, United Kingdom). In the final step, 10 ng/ml of recombinant mouse stem cell factor SCF (R&D systems, MN, United States) and 5 ng/ml recombinant murine IL-3 (R&D Systems, MN, United States) were added. The cells were cultured in 7.5% CO_2_ at 37°C for 4 weeks until they differentiated into BMMCs. Prior to use in experiments, cells from each preparation were analyzed for surface expression of FcεRI and SCF receptor (c-kit), the classic mast cell markers, by flow cytometry. Only cultures in which >95% viable cells stained positive for both c-kit and FcεRI were used.

### Dorsal Root Ganglion (DRG) Culture

Dorsal Root Ganglion were isolated and cultured according to previously described procedure (Sleigh et al., 2016). DRGs isolated from adult (8–12 week old) C57BL male mice, were dissociated with 0.06 μg/ml collagenase XI (Sigma) and 0.1 μg/ml Dispase for 1 h at 37°C, followed by gentle trituration. For selective isolation of neurons, gradient centrifuge technique with 15% bovine serum albumin (BSA) in medium was used. Cells were cultured in complete Neurobasal-A medium (NBA, Gibco) containing 2% B-27 supplement (Gibco), 2 mM Glutamax (Gibco), 1% penicillin/streptomycin (Gibco), 10 ng/ml NGF (Sigma) and 1 μM Cytosineβ-D-arabinofuranoside (Ara-C, Sigma) and seeded on 16 mm matrigel (BD) – coated glass coverslips or 96 well flat bottom plates and incubated for 1 day before using in co-culture.

### BMMC-DRG Co-culture

After culturing BMMC for 4 weeks, the purity of mast cells was assessed for surface expression of FcεRI and c-Kit by flow cytometry. Only BMMC cultures with >95% FcεRI^+^ and c-Kit^+^ were used for co-culture. 1–3 × 10^5^ BMMCs suspended in co-culture medium (50% IMDM and 50% NBA) were added to DRG cultures prepared 24 h previously. Co-cultures were incubated in 37°C with presence of IL-3 (5 ng/ml) for different time points. For some experiments, DRG were preincubated for 30 min prior to co-culture with 1–30 μg/ml of CADM1 blocking peptide (9D2, Medical & Biological Laboratories). For separation experiments, transwells (Costar, Corning) with a 0.4-μm insert were used. DRG were cultured in the lower chamber, while BMMCs were added in the insert.

### BMMC Sensitization, Degranulation and Cytokine Secretion Assay

For antigen stimulation experiments, BMMCs (3.5 × 10^5^ cells) were sensitized overnight with 0.5 μg/ml anti-dinitrophenyl IgE (anti-DNP IgE, Sigma-Aldrich, United Kingdom). On the following day, IgE-presensitized BMMCs were co-cultured with DRG for various time points and then stimulated by 10–100 ng/ml of dinitrophenyl antigen (DNP) (Sigma-Aldrich, United Kingdom) for 30 min at 37°C. The co-culture supernatant was collected and analyzed for degranulation, IL-6, and TNFα secretion.

Degranulation of mast cells was evaluated by measuring the activity of granule-stored enzyme β-hexosaminidase (β-hex) release (Gilfillan and Tkaczyk, 2006). After BMMC were activated by IgE/Ag cross-linking, supernatants were collected and incubated with the same volume of substrate solution [2 mM *p*-nitrophenyl *N*-*acetyl β-*D-glucosamine (Sigma-Aldrich, United Kingdom) in 100 mM citrate buffer (pH 4.5)] at 37°C for 2 h. The reaction was stopped by addition 90 μl of Tris–HCL (pH 9). Enzyme activity was evaluated by measuring optical density at 405 nm with a microplate reader (OPTIMA). The total amount of β-hex released was determined by cell lysis with 0.5% Triton X-100. Background absorbance readings (b) were determined from wells containing all buffers except supernatant. The β-Hex activity was calculated using the following formula: degranulation (%) = ((supernatant-b)/(Total-b)) × 100. Fold change of enhancement in degranulation was calculated by dividing the percentage of degranulation in tested condition to the percentage of degranulation in the control condition.

For cytokines production, the experiment was performed as above, but BMMCs were stimulated with DNP for 6 h at 37°C. IL-6 and TNFα were analyzed in supernatants by mouse IL-6 ELISA kit (R&D Systems) and mouse TNFα ELISA kit (R&D Systems), respectively, as per manufacturer’s instructions.

### Fluorometric Calcein-Adhesion Assay

Adhesion of BMMC was assessed using Calcein Cell Adhesion Assay Kit (Invitrogen, Life Technologies) as previously described (Moiseeva et al., 2013a). Following manufacturer’ protocol, 1 × 10^6^ BMMCs/ml of serum-free medium were labeled with 5 μM Calcein-AM for 30 min at 37°C. Then, calcein-labeled BMMCs were resuspended with co-culture medium at a density of 1 × 10^5^ BMMCs/100 μl/well and co-cultured with DRG for 2 h. Un-attached BMMCs were washed out by spinning the plate upside down at 20 × *g* for 2 min and wells were re-filled with co-culture medium. The fluorescent signal was measured by fluorescence plate reader before and after washing step for total and attached readings, respectively. Calcein excitation wavelength used was 485 nm and emission wavelength was 520 nm. Adhesion was measured as percentages of adherent BMMCs to the total. For comparison control, calcein-labeled BMMCs were seeded on 1-day old Matrigel-coated wells in same co-culture medium and for the same time like the ones with DRG co-culture.

### Flow Cytometry

For BMMC surface protein expression, 1 × 10^6^ BMMCs/sample were used. After washing BMMCs with cold PBS, cells were re-suspended in cold FACS buffer (2 mM EDTA in PBS with 2% FBS). BMMCs were blocked with 1:100 of Fc Block (CD16/32) (eBioscience) for 15 min on ice to prevent non-specific binding of antibodies. After washing with FACS buffer twice, BMMCs were incubated with APC-anti-mouse c-Kit (eBioscience, 1:100) and PE-anti-mouse FcεRI (eBioscience, 1:100), when checking for mast cell differentiation, or rabbit anti-CADM1 (Santa Cruz sc-33198, 1:100) antibodies for 20 min on ice. Alexa Flour^®^488 anti-rabbit (Invitrogen, 1:1000) was used as secondary antibody. Fluorescence was detected with FACSCalibur (BD Biosciences) at emission wavelengths of 488 nm for Alexa Fluor^®^488 labeled samples, 660 nm for APC labeled samples or 585 nm for PE labeled samples. For analysis, the viability was first gated based on side scatter and electronic volume from unstained sample. Only viable cells from gated population were included in fluorescence measurements. For CADM1 intracellular expression, BMMCs were fixed in 4% paraformaldehyde in PBS (pH 7.4) for 20 min at 4°C. Then, permeablized with 0.1% Triton X-100 for 10 min.

### Immunocytochemistry

Cultured cells were washed with ice-cold PBS and fixed with 4% PFA for 10 min, and permeabilized with 0.1% Triton X-100 in PBS for 10 min. Following 1 h incubation with blocking buffer (2% normal donkey serum (Sigma), 0.2% Fish serum gelatine (FSG) Sigma, G7765) and 0.01% Triton X-100) in PBS) at room temperature, cells were stained with primary antibodies and incubated overnight at 4°C. Cells were then washed three times with PBS and stained with Alexa Fluor^®^anti-mouse or anti-rabbit secondary antibodies (1:1000 Invitrogen) for 2 h in the dark at room temperature. Glass coverslips were mounted onto microscope slides using mounting medium (Vectashield Hard set H1500, Vector) with 4′,6-diamidino-2-phenylindole (DAPI) to stain the nucleus. For negative control, some wells were stained with only secondary antibodies. Images were viewed using a 40× and 60× oil objective (N.A. 1.42) on Nikon A1 confocal microscope. Samples were illuminated at the required wavelength using 405, 488, and 561 nm lasers.

Primary antibodies used for immunocytochemistry were as follows: Rabbit anti-peripherin (Sigma P5117, at 1:1000), Guinea pig anti-Substance P (Abcam ab10353, 1:100), Mouse anti-CGRP (Abcam Ab81887, 1:100), mouse anti-β III Tubulin monoclonal IgG (R&D MAB1195 clone TuJ-1 lot HGQ0113121, 1:1000), Rabbit Anti-CADM1 Polyclonal IgG (H-300) (Santa Cruz sc-33198 lot F0407, 1:300), Alexa Fluor^®^488 anti-mouse c-Kit (Biolegend (6861), 1:100), Mouse anti-Tryptase (Abcam ab2378, 1:300). For further details please see Supplementary Table 1.

### Western Blot

A total of 1 × 10^6^ of 4-week old BMMCs or one-day old DRG cultures were lysed in ice-cold lysis buffer (50 mM Tris–HCl, 150 mM NaCl, 0.3% Triton X-100, pH 8) with 1% of protease inhibitor cocktail III (Fisher Scientific). The insoluble debris was removed by spinning at 14,000 g for 20 min at 4°C. The protein concentration of each lysate was determined using Bradford protein assay (Sigma). For separation of CADM1 protein, 10% SDS resolving gel was used. After running the gel, the protein transfer was performed at 85 V for 90 min at 4°C (protein of interest 110 kDa). Then, the membrane was probed using rabbit anti-mouse CADM1 antibody (1:300, Santa Cruz) and mouse anti-GAPDH (1:5000, Thermo Fisher Scientific) and visualized using Li-cor system, Goat IRDye 800 anti-Rabbit 1:5000 and Goat IRDye 700 anti-Mouse 1:5000.

### Amaxa Nucleofection of BMMC

Nucleofector II (Amaxa, Lonza) was used to knockdown CADM1 in BMMC. Basic fibroblasts nucleofector kit (VPI-1002, 90279050) and recommended programs (X-001) was optimized for BMMC transfection. 4-5 × 10^6^ BMMCs/reaction were transfected with 2 μg of psi-U6 plasmid (Genecopoeia) expressing CADM1 ShRNA and eGFP or scrambled ShRNA and eGFP. Transfected cells were incubated with complete IMDM for 48 h at 37°C and 7.5% CO_2_ before sorted using fluorescence-activated cell sorting (FACS). Only the GFP expressing cells were used for subsequent experiments. Three unique constructs of CADM1 ShRNA plasmids and non-targeting scramble control (Genecopoeia) were tested separately to identify the most efficient construct. CADM1 -ShRNA1 MSH031688-31 (ggacagaatctgtttactaaa), CADM1- ShRNA2 MSH031688-32 (cctccacgtaacttgatgatc), CADM1- ShRNA3 MSH031688-33 (ggagattgaagtcaactgtac), Scramble- ShRNA CSHCTR001.

### Statistical Analysis

The results are expressed in the figures as the means ± standard error of the mean (SEM) of at least three independent experiments based on different mouse cultures. Statistical comparisons with the appropriate control data from adhesion assay experiment with blocking peptide and all knockdown experiments were performed using one-way repeated measures (ANOVA) followed by Turkey’s post-test. Data from other experiments were analyzed using paired *t*-test. Probability values (*p*) < 0.05 were considered statistically significant. All data handling, statistical analysis, and graphs were prepared using GraphPad Prism (GraphPad Software, La Jolla, CA, United States)^1^.

## Results

### CADM1 Is Expressed in Mast Cells and Sensory Neurons

**FIGURE 1:**
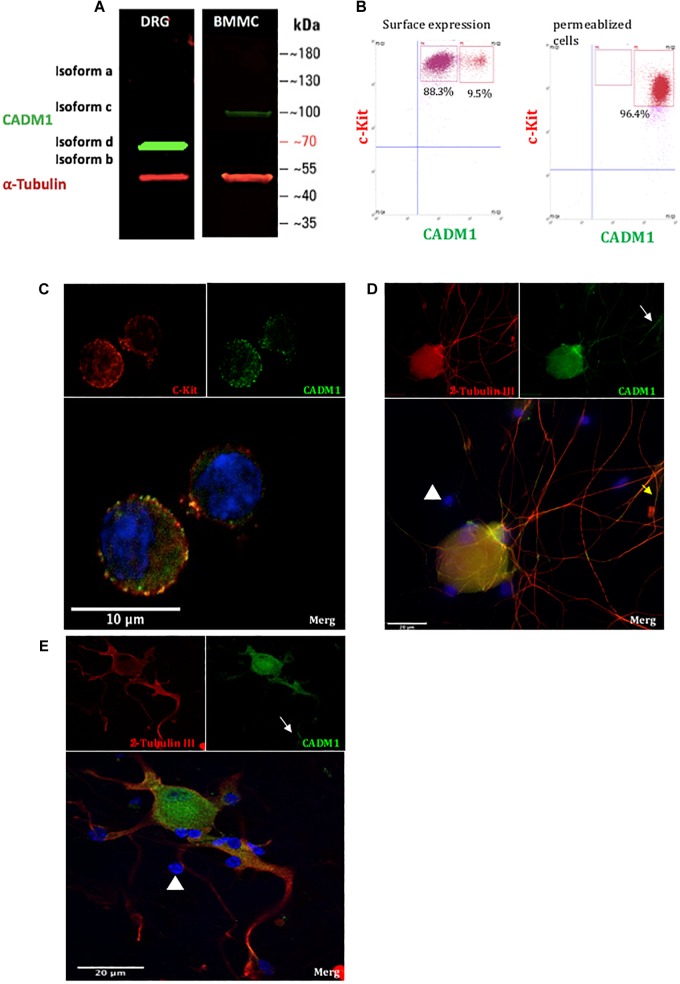
CADM1 is expressed in BMMCs and sensory neurons. **(A)** Immunoblot of lysates prepared from DRG and BMMC mono-cultures probed with anti-CADM1 and anti-α-Tubulin, as a loading control. The m.w. scale is shown to the right of the blot. **(B)** Flow cytometric analysis of surface (left) and total CADM1 expression in Triton X-100-permeablized BMMCs (right). BMMCs were double labeled with conjugated c-Kit and CADM1 Abs. BMMCs were gated as c-kit+ CADM1high cell subset and c-kit+ CADM1medium cell subset. Numbers in plots are the percentages of cells in the indicated gate. **(C)** Confocal immunofluorescence images of c-Kit (red), CADM1 (green), and DAPI (blue) and merged image of mono-cultured BMMCs. Scale bar represents 10 μm. **(D,E)** Confocal immunofluorescence images of β-Tubulin (red), CADM1 (green) and DAPI (blue) in mono-cultured DRGs. White arrows indicate CADM1 immunoreactive neurites. Note that non-neuronal cells, observed as DAPI positive, β-Tubulin negative are also negative for CADM1 (arrowheads). Scale bar represents 20 μm.

**FIGURE 2:**
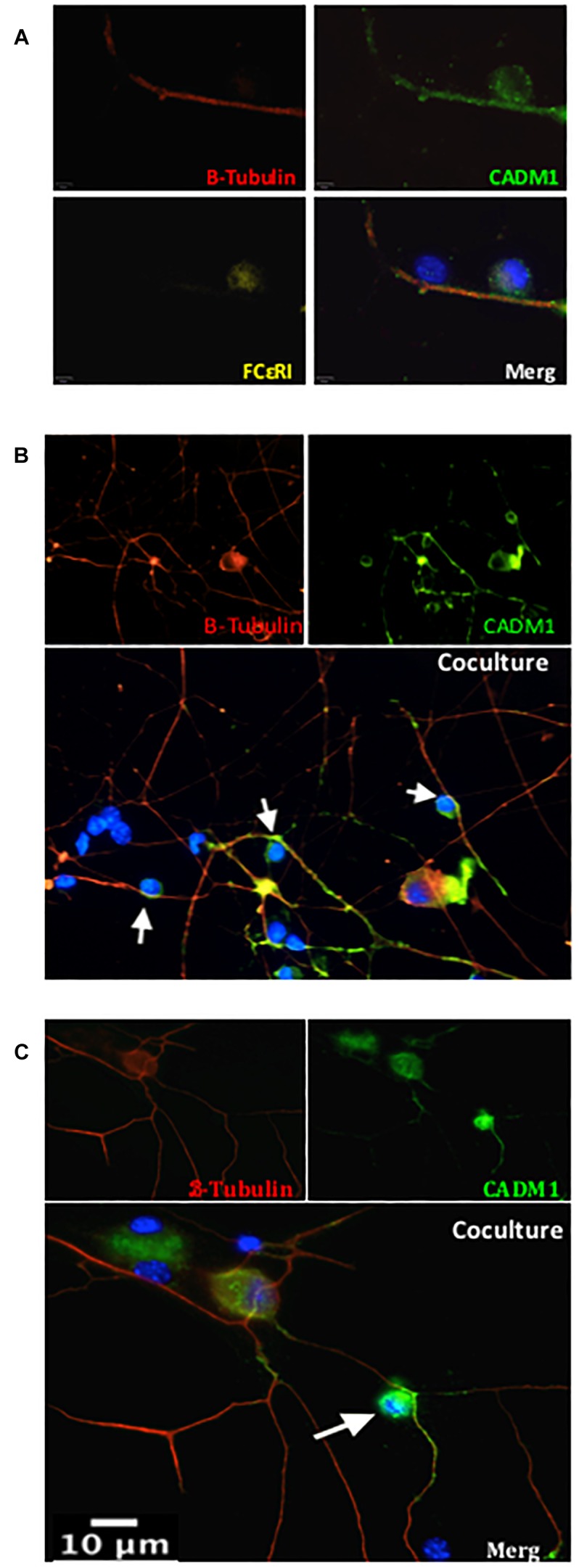
CADM1 is enriched at mast cell-sensory neurite contact sites. Representative immunofluorescent images of BMMCs co-cultured with DRG neurons for 24 h. In panel **(A)** individual and overlaid images are shown from a neurite with two adherent cells. β-Tubulin (red) is used as the neuronal marker, DAPI (blue) nuclear marker labels all cells in the culture, while FcεRI (yellow) specifically labels BMMCs. CADM1 (green) stained positive in the sensory neuron neurite and BMMC, but is absent from the attached non-neuronal, non-mast cell. **(B,C)** Representative individual and overlaid immunofluorescent images of CADM1 (green), β-Tubulin (red) and DAPI (blue) staining of different co-cultures. Increased CADM1 fluorescent intensity at contact sites (white arrows) between BMMCs and neurites (red).

As a prelude to understanding the role that cell adhesion plays in regulating mast cell-sensory neuron interactions, we established a co-culture system of C57BL6 mouse BMMCs and primary DRG neurons isolated from adult mice (8–12 weeks). The purity of neurons used in the co-cultures was estimated to be 30%, as quantified by immunohistochemistry of anti-β tubulin positively stained cells relative to the total number of DAPI positively stained cells. Prior to co-culture, BMMCs were differentiated for 4 weeks with IL-3 and rm-SCF and assessed for purity and maturity by flow cytometry and their ability to undergo antigen-induced degranulation. Only BMMC cultures in which >99% of cells stained positive for expression of c-Kit and FcεRI, well established markers of mature mast cell, were used in co-culture experiments. The expression and distribution of CADM1 in each cell type before and after 48 h co-culture was then examined. Western blot analysis of lysates prepared from pure BMMCs exhibited a single band for CADM1 with a molecular weight of ∼100 kDa (Figure 1A), consistent with previous reports for human and mouse mast cells (Furuno et al., 2005; Moiseeva et al., 2013a). Immunocytochemistry and flow cytometry showed moreover that CADM1 was predominantly located in the plasma membrane of BMMCs (Figure 1B,C). CADM1 expression was also observed in lysates prepared from adult DRG cultures, although it had a lower molecular weight (∼70 kDa, Figure 1A), consistent with the expression of a distinct splice variant lacking *O*-glycans (Hagiyama et al., 2011). Immunocytochemistry of DRGs maintained in mono-culture for 48 h with the neuronal marker β-tubulin confirmed the expression of CADM1 was specific to neurons and notably absent from non-neuronal glia-like cells which surrounding the neurons, whose presence can be detected from DAPI staining of their nuclei (Figure 1D,E). Close inspection of CADM1 staining in the co-cultures showed that it was most intense at the sites of contact between mast cells and neurites (Figure 2 arrows).

### CADM1 Is Necessary for BMMC Adhesion to DRG Neurons

**FIGURE 3:**
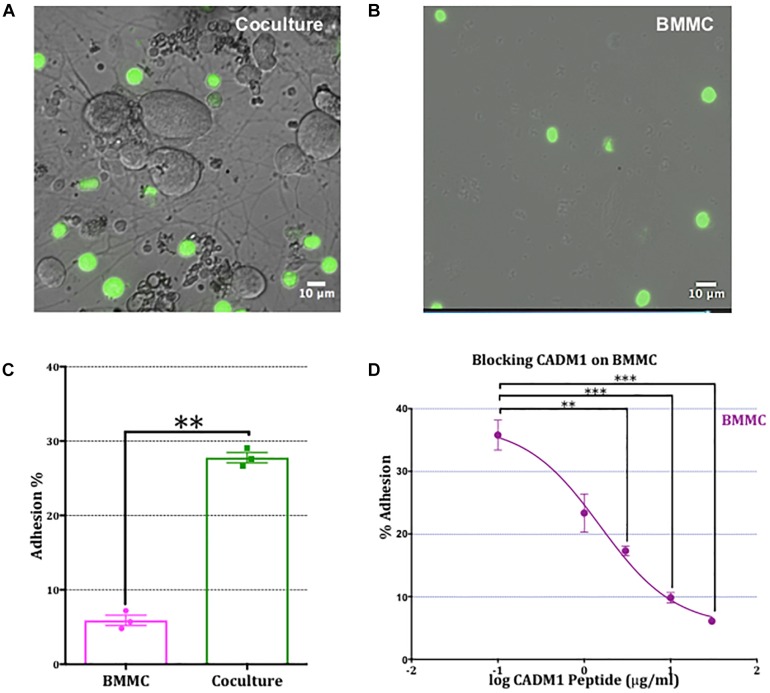
Adhesion of mast cells to sensory neurons is attenuated by a CADM1 blocking peptide. **(A)** Superimposed bright-field and fluorescent image of live calcein-labeled, adherent BMMCs (green) co-cultured with DRG neurons (unlabeled cells) for 2 h. Non-adherent cells have been removed by washing and centrifugation of the plate. **(B)** Image of calcein-labeled BMMCs plated in parallel into matrigel-coated wells devoid of neurons and subjected to the same washing and centrifugation procedure. **(C)** BMMC adhesion quantified from calcein-fluorescence remaining in wells after washing and centrifugation expressed as a percentage of total well fluorescence measured prior to washing procedure. Data shown as mean ± SEM from *N* = 3. Each done in duplicate. Data were analyzed using a two-tailed paired *t*-test ^∗∗^*p* < 0.01. **(D)** Concentration-dependent inhibition of mast cell adhesion to DRG measured with a CADM1 blocking peptide. Percentage of adherent BMMC was calculated using the calcein adhesion assay. Each condition was done in duplicate, on *N* = 3 cultures. Each point represents the mean ± SEM. One-way ANOVA followed by Turkey’s multiple comparison post-test was performed. ^∗∗^ denotes *p* < 0.01 and ^∗∗∗^ *p* < 0.001 compared to the percentage of BMMC adhesion in the absence of CADM1 blocking peptide.

To assess the role of CADM1 in mediating BMMC adhesion to sensory neurons, we developed a fluorimetric adhesion assay based on labeling of BMMCs with calcein, prior to their addition to DRG cultures. Optimization experiments showed that after loading BMMCs with calcein-AM for 30 min, fluorescent labeling is stable for 3 h and subsequently declines over the next 24 h. Isolated adult DRG neurons were cultured for 24 h in a flat-bottomed 96 well tissue culture plate during which time they developed an extensive network of neurites (Figure 3A). Calcein-labeled BMMCs were then added to the wells and allowed to adhere for 2 h after which non-adherent cells were removed vigorously by centrifugal spinning the culture plate upside-down at 20 × *g* for 2 min. To control for non-specific adhesion of BMMCs to the matrigel matrix used for supporting the DRG cultures, experiments were done in parallel on wells coated with matrigel but devoid of DRG (Figure 3B). Microscopic examination of the wells after spinning, clearly shows the enhancement in BMMC adhesion induced by co-culture with neurons, and moreover that the majority of mast cells attach to neurites rather than cell bodies (Figure 3A), consistent with observations made during the immunohistochemistry experiments shown in Figure 2. To quantify BMMC adhesion to the neurons, we measured the total fluorescence per well from calcein-labeled BMMCs before (total) and after centrifugation (for adherent). As shown in Figure 3C, co-culture with DRG neurons significantly increased the number of adherent BMMCs four-fold. Addition of CADM1 blocking peptide inhibited adhesion of BMMCs to sensory neurons in a concentration-dependent manner (Figure 3D) and was almost abolished at the maximum concentration tested (30 μg ml^-1^), consistent with the hypothesis that CADM1 mediates adhesion between mast cells and sensory neurons.

**FIGURE 4:**
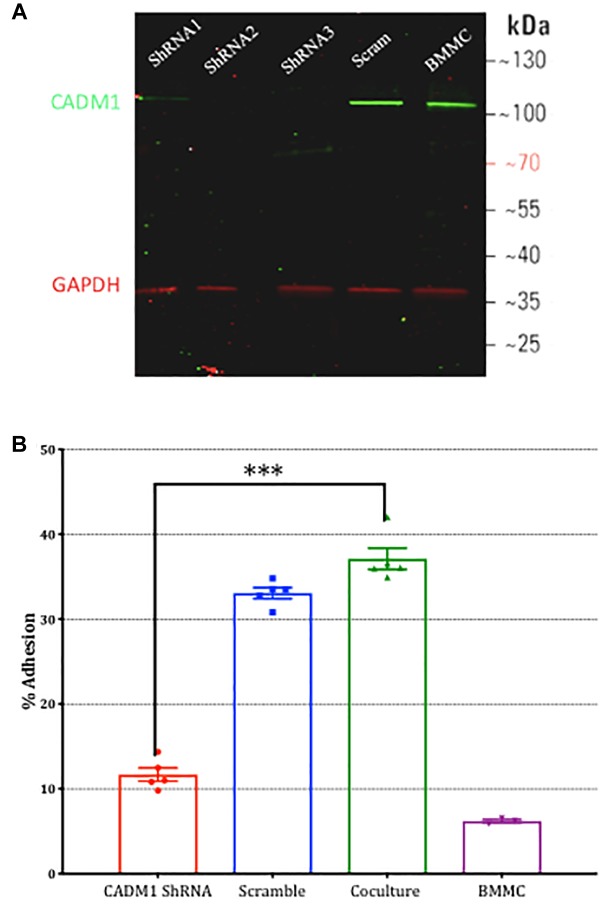
Knockdown of CADM1 in mast cells inhibits their adhesion to sensory neurons. **(A)** Western blot analysis of CADM1 expression in BMMCs transfected with the indicated ShRNA 48 h previously. Expression from un-transfected control cells examined in parallel are also shown (lane labeled BMMC). GAPDH was used as loading control. Data are representative of three independent experiments. **(B)** Adherence of BMMCs transfected with CADM1 ShRNA or scrambled control to neurons tested using the calcein assay. BMMC adhesion in the absence of DRG neurons is shown as a control. CADM1 knockdown significantly reduced BMMC adhesion to sensory neurons (*N* = 3). Each bar represents the mean ± SEM. One-way ANOVA followed by Turkey’s multiple comparison post-test was performed. ^∗∗∗^ denotes *p* < 0.001.

To validate the results from the CADM1 blocking peptide, we also performed CADM1 knockdown experiments in BMMCs. Three vectors expressing unique CADM1 targeted shRNA or a non-targeting scramble control together with an eGFP reporter gene were transfected into BMMCs. After 48 h, cells were sorted using fluorescence activated sorting and eGFP expressing cells used in subsequent experiments. The efficacy of CADM1 knockdown in sorted eGFP expressing BMMCs as assessed by western blot (Figure 4A) showed that all three ShRNA constructs tested were highly effective, with ShRNA2 and ShRNA3 achieving almost complete knockdown. Consistent with the results from the CADM1 blocking peptide experiments, knockdown of CADM1 expression in BMMCs significantly attenuated their adhesion to sensory neurons (Figure 4B).

### CADM1-Dependent Adhesion to Sensory Neurons Potentiates Antigen-Induced Mast Cell Degranulation and Cytokine Secretion

**FIGURE 5:**
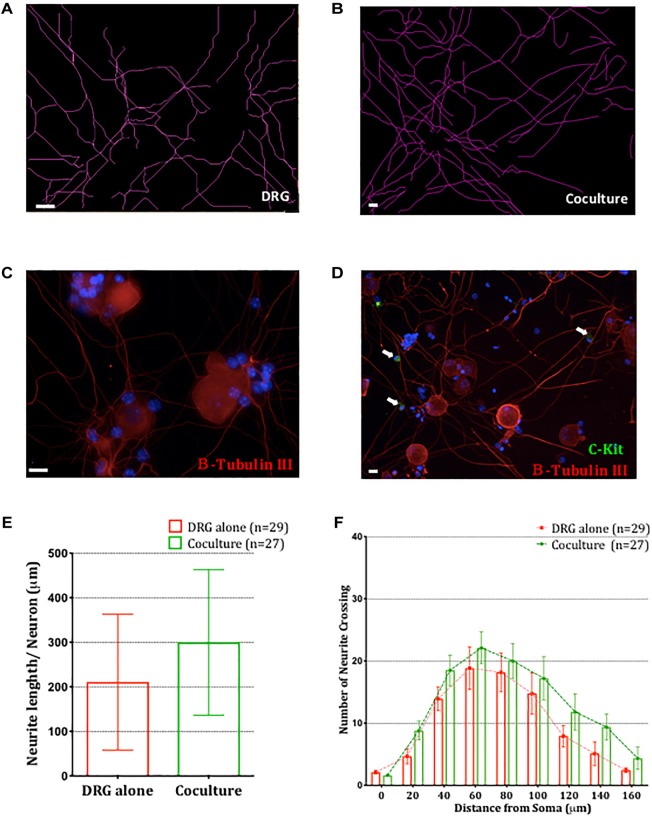
Co-culture of BMMCs with DRG does not impact neuronal morphology. Representative schematic tracings of the neurites from DRG cultured alone **(A)** or in BMMC-DRG co-culture **(B)**. Soma and neurites were visualized with anti-β-III tubulin (red) and BMMCs with anti-c-kit (green, white arrows) in DRG mono-cultures **(C)** and co-cultures **(D)**. DAPI staining of nuclei (blue) show presence of non-neuronal cells in the DRG cultures. **(E)** Quantitative analysis of total neurites length in DRG mono-cultures and co-cultures, compared using unpaired *t*-test. **(F)** Sholl analysis of the neurite complexity. Each bar represents the mean ± SEM of number of crossing neurites found in each given distance from the soma. Statistical analysis using multiple *t*-test. *N* = 3.

Having established that BMMCs adhere to sensory neurons via a CADM1-dependent interaction, we next examined potential functional consequences of this interaction. It has been reported that the length of substance P immuno-reactive nerve fibers are increased in airway of allergic conditions such as asthma (Ollerenshaw et al., 1991) and that NGF and TNFα secreted by activated mast cells could enhance neuronal outgrowth (Leon et al., 1994; Kakurai et al., 2006). Immunocytochemical analysis of DRG cultures used for our co-cultures showed they consisted of at least 60% nociceptors, of which ∼50% were peptidergic (Supplementary Figure 1). We therefore investigated the effect of co-culturing BMMC on sensory neuron morphology in our *in vitro* system. Two parameters examined were total neurite length and complexity (number of neurite crossing points of a concentric circle set with radii increasing by 20 μm, (Stanko et al., 2015) and comparisons made between DRG monocultures and BMMC-DRG co-cultures. The neurites and BMMCs were detected by immunocytochemistry staining for βIII-tubulin and c-kit, respectively (Figure 5A–D). After 48 h of co-culture, no significant change in neurite length nor complexity was detected (Figure 5E,F), indicating that at least in the short term (48 h post-axotomy), mast cell adhesion does not alter sensory neurite morphology.

**FIGURE 6:**
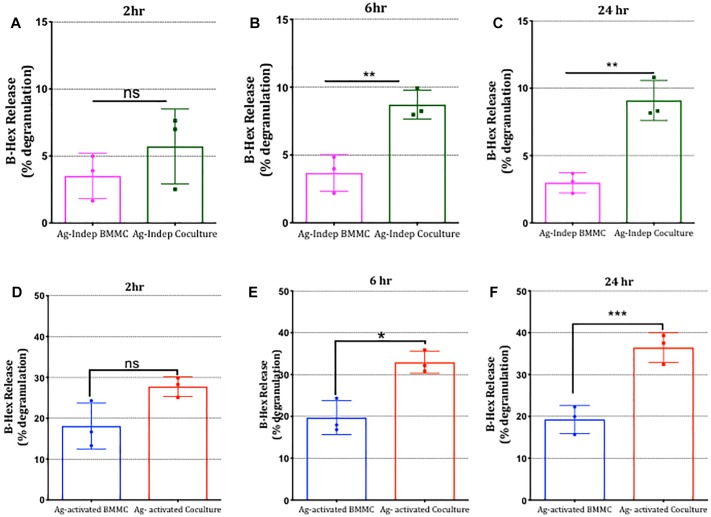
Mast cell degranulation is potentiated following co-culture with sensory neurons. BMMCs pre-sensitized with anti-DNP IGE were cultured alone or with DRG for various times as indicated **(A–F)**. Degranulation measured as secreted β-hexosaminidase (β-hex) in media recovered from the cultures was measured at the times indicated above each graph in the absence **(A–C)** or after 30 min exposure to the antigen (Ag) DNP (10°ng/ml, **D–F**) and expressed as a percentage of total β-hex measured from BMMC lysed with 0.5% Triton X-100. Data shown are mean ± SEM of *N* = 3, each performed in duplicate. ^∗^*p* < 0.05, ^∗∗^ *p* < 0.01 and ^∗∗∗^ *p* < 0.001 compared to BMMC alone. Data were analyzed using two-tailed paired *t*-test.

To investigate whether adhesion of mast cells to sensory neurons alters mast cell function and more specifically, antigenic activation of mast cells through FcεRI receptors, we established co-cultures of DRG neurons with anti-DNP IgE-sensitized BMMCs and compared their responses to antigen stimulation with mono-cultures of BMMCs prepared in parallel. Degranulation of mast cells was measured using β-hexosaminidase (β-hex) assays (Kuehn et al., 2010). Remarkably, BMMC basal degranulation and antigen-stimulated degranulation were both significantly potentiated in BMMCs following 6 h co-culture with sensory neurons and continued to increase for up to 24 h, the latest time point tested (Figure 6). Indeed after 24 h of co-culture with sensory neurons, mast cell degranulation was approximately double that measured in mono-cultures set up in parallel. In contrast, no such potentiation of antigen-induced degranulation was observed when BMMCs in co-culture were stimulated with compound 48/80 (Supplementary Figure 2), indicating that the effect of co-culture was not simply making the mast cells hyper-responsive in a non-specific manner but involved a specific signaling pathway which enhanced their responsiveness to antigen-stimulation in a time-dependent manner.

**FIGURE 7:**
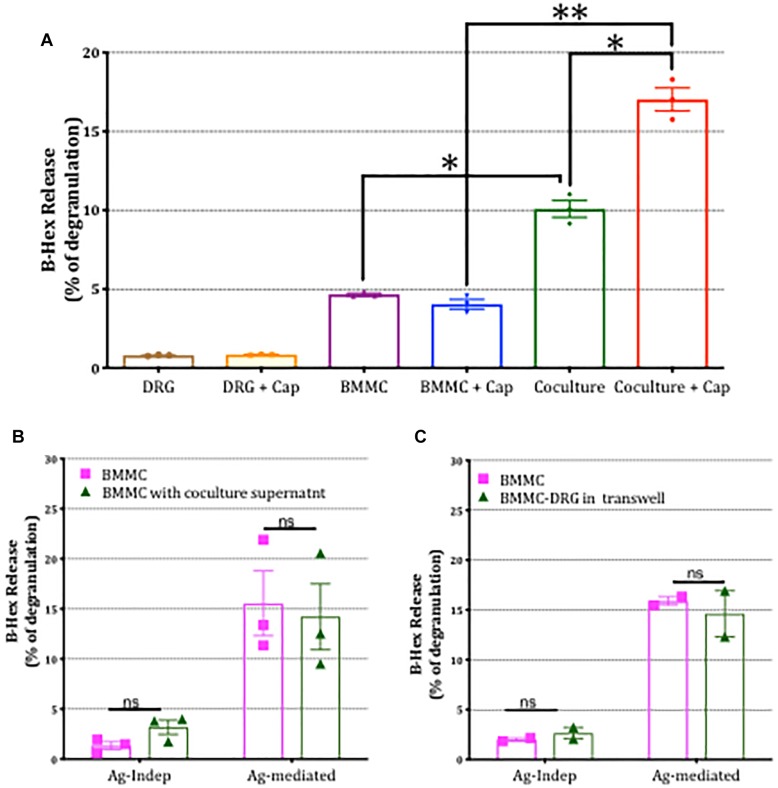
Potentiation of mast cell degranulation by sensory neurons is contact-dependent. **(A)** BMMC degranulation measured as secreted β-hex from the mono-cultures and co-cultures of the indicated cells. Sensory neurons co-cultured with BMMCs for 24 h, were activated with 1 μm capsaicin for 20 min. β-Hex secreted from mono-cultures of DRGs is minimal and shows that the potentiated BMMC degranulation observed in the co-cultures is not simply additive but due to a significant interaction between the two cell types. **(B)** BMMCs pre-sensitized with anti-DNP IGE were cultured alone or with supernatant collected from BMMC-DRG co-cultured for 24 h. Degranulation as measured by secreted β-hex was measured in resting condition (labeled Ag independent) or after 30 min stimulation with the antigen, DNP (10 ng/ml, labeled Ag-mediated), and expressed as a percentage of total β-hex measured from lysed BMMCs. **(C)** Pre-sensitized BMMCs were cultured alone or on the top of DRG cultured using a transwell system for 24 h prior to measuring degranulation. Data shown are mean ± SEM of *N* = 3, each performed in duplicate. Data were analyzed using two-tailed paired *t*-test. ^∗^*p* < 0.05, ^∗∗^*p* < 0.01, n.s is non-significant.

To examine the possibility that mediators released by DRG neurons in co-culture mediated the enhancement of mast cell degranulation, we performed three different types of experiments. Firstly we examined the impact of stimulating sensory neurons directly with capsaicin on mast cell degranulation. As shown in Figure 7A, BMMC degranulation in co-cultures was significantly potentiated by capsaicin showing that chemical communication between sensory neurons and mast cells was functional under co-culture conditions. Next we examined the impact of disrupting contact between the two cell types by (a) incubating BMMCs for 24 h with supernatant from BMMC-DRG co-cultures, and (b) preparing co-cultures of BMMCs and DRGs in which contact between the two cell types was blocked by means of a transwell insert. In either scenario, where direct contact between sensory neurons and mast cells was disrupted, the potentiation of mast cell degranulation by sensory neuron signaling was blocked (Figure 7B,C) emphasizing the need for adhesion between the two cell types.

To explore further the specificity of the functional interaction between mast cells and sensory neurons and its dependence on CADM1-mediated adhesion, we also examined the effects of co-culturing BMMCs with HEK cells. Like sensory neurons, HEK cells also express CADM1 and BMMCs adhere to these cells (Supplementary Figure 3). However, co-culture of BMMCs with HEK cells did not result in potentiation of mast cell degranulation (Supplementary Figure 3), the percentage of basal degranulation measured being 5.8 ± 1.6% in BMMC-HEK co-cultures maintained for 24 h, compared with 6.5 ± 0.7% for BMMC mono-cultures set up in parallel (*N* = 3). Antigen-induced degranulation was similarly unaffected by co-culture with HEK cells (co-culture degranulation 17.9 ± 1.8%, compared with 18.2 ± 1.6% for BMMC mono-cultures set up in parallel, *N* = 3, Supplementary Figure 3).

**FIGURE 8:**
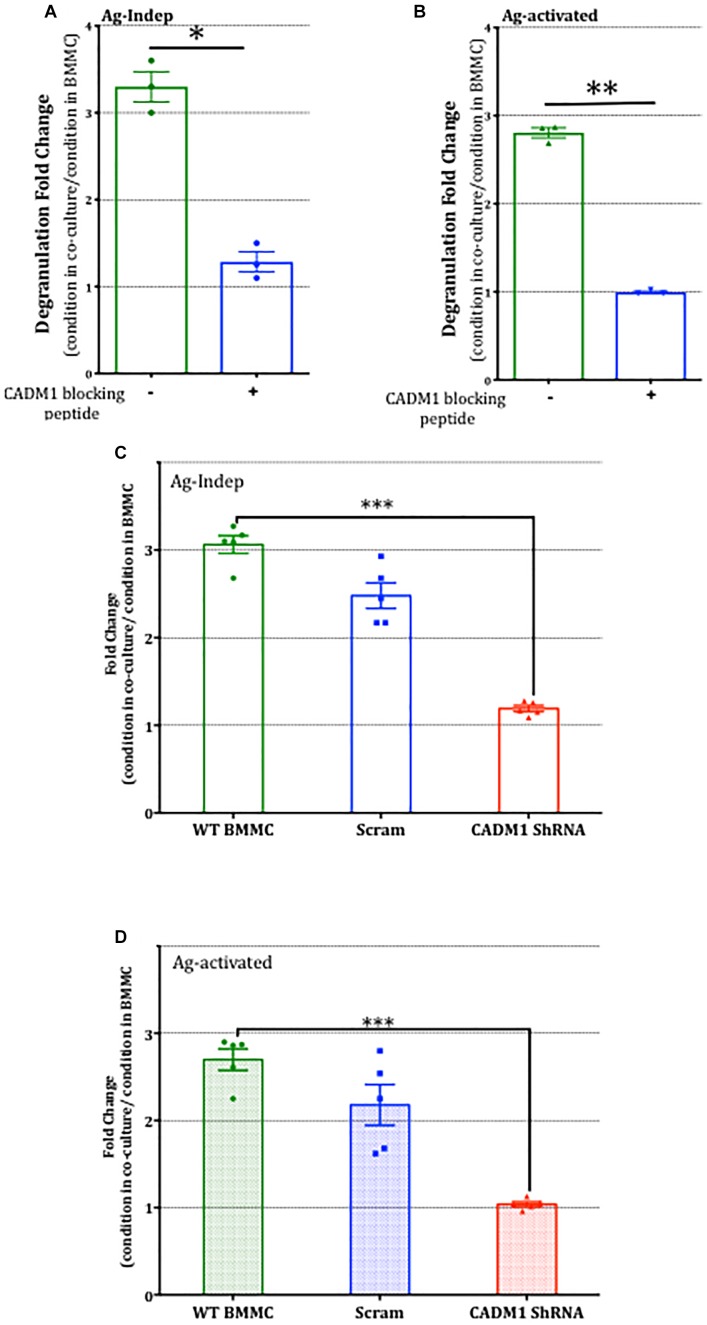
CADM1 is necessary for mast-cell sensor neuron cross-talk. BMMCs pre-sensitized with anti-DNP IGE were co-cultured with DRG for 24 h in the presence or absence of 10 μg/ml CADM1 blocking peptide before degranulation was quantified using the β-Hex assay. Data were normalized to degranulation measurements made from BMMC mono-cultures set up in parallel, and the fold-change in basal Ag-Indep degranulation **(A)** or DNP (10 ng/ml), Ag-activated degranulation **(B)** calculated and compared by two-tailed paired *t*-test, ^∗^ denotes *p* < 0.05, ^∗∗^*p* < 0.01. Each bar represents the mean ± SEM (*N* = 3). **(C)** Untransfected (WT BMMC), CADM1-ShRNA or scramble-transfected BMMCs pre-sensitized with anti-DNP IGE were co-cultured with DRG for 24 h before degranulation was quantified using the β-Hex assay. Fold change of Ag-Independent degranulation **(C)** and DNP (10 ng/ml) Ag-activated degranulation **(D)** were calculated and compared by one-way ANOVA followed by Turkey’s multiple comparison post-test, ^∗∗∗^denotes *p* < 0.001 compared to wt BMMC-DRG co-culture. Each bar represents the mean ± SEM (*N* = 3).

Having established that potentiation of BMMC degranulation was specific to co-culture with DRG and reliant on contact, we next examined the role of CADM1-dependent adhesion in mast cell-sensory neuron crosstalk. Addition of CADM1 blocking peptide to the co-cultures was found to significantly inhibit the potentiation of mast cell degranulation (Figure 8A,B), indicating that CADM1-mediated adhesion between the cell types was necessary and sufficient to potentiate mast cell secretion. Consistent with this conclusion, knockdown of CADM1 in BMMCs also significantly attenuated the enhancement of degranulation induced by co-culture with sensory neurons (Figure 8C,D).

**FIGURE 9:**
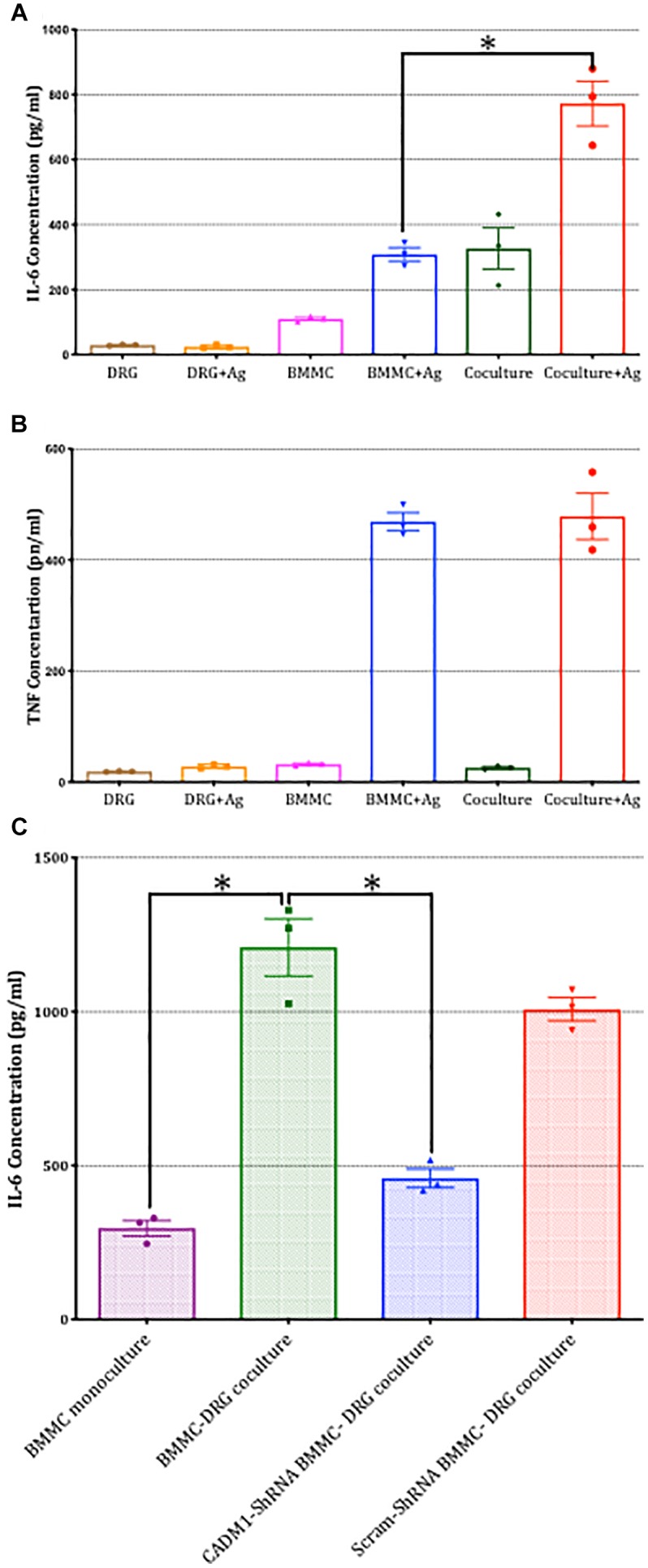
Sensory neuron potentiation of antigen-stimulated cytokine secretion by mast cells is dependent on CADM1. BMMCs pre-sensitized with anti-DNP IGE were cultured alone or with DRG for 24 h. **(A)** Secreted IL-6 and **(B)** TNFα were quantified by ELISA for cells stimulated in parallel with the antigen DNP (10 ng/ml) for 6 h or vehicle control. Data shown are mean ± SEM of *N* = 3, each performed in duplicate. ^∗^ *p* < 0.05 compared to stimulated BMMC alone. Data were analyzed using two-tailed paired *t*-test. **(C)** BMMC mono-culture and untransfected WT, CADM1-knockdown or scramble-transfected BMMCs were co-cultured with DRG for 24 h, prior to being activated with DNP (10 ng/ml) for 6 h. IL-6 was measured from supernatants and compared by one-way ANOVA followed by Turkey’s multiple comparison post-test, ^∗^denotes *p* < 0.05 compared to wt BMMC-DRG co-culture. Each bar represents the mean ± SEM (*N* = 3).

Finally, we examined whether CADM1-dependent mast-cell sensory neuron cross talk extended to the regulation of pro-inflammatory cytokine secretion. IL-6 and TNFα secretion in mono- and co-cultures were compared in the absence and presence of antigen activation of mast cells. As shown in Figure 9A, IL-6 secretion was induced by co-culture with DRG and moreover, antigen-activated secretion significantly increased by 2.5-fold. In contrast, TNFα was not increased, indicating that the signaling pathway enhanced by CADM1 adhesion of mast cells to sensory neurons was specific and selective to the synthesis and secretion of specific pro-inflammatory cytokines. Control experiments confirmed that neither cytokine was produced to any significant level in DRG monocultures (Figure 9A,B). Knockdown of CADM1 expression in BMMCs significantly attenuated the neuronal induced IL-6 secretion as well as the neuronal enhancement of antigen-activated IL-6 secretion, confirming the critical role played by this adhesion molecule in regulating the signaling pathway controlling cytokine expression and secretion in mast cell-sensory neuron cross talk (Figure 9C).

## Discussion

Because mast cells mature within tissues when they are in proximity to other cell types (Galli, 2000), one can speculate that mast cell specific interactions with other cells will be regulated by the expression of cognate adhesion receptors on other cells in the tissue and that contact between the cells will promote receptor mediated communication between the cells. Furthermore, anchoring of adhesion molecules at sites of cell contact and consequent stabilization of protein-protein interactions mediated through their cytosolic domains may also influence the effector functions of mast cells in a tissue and cell specific manner. Here we show, that CADM1 mediates adhesion between mast cells and adult sensory neurons, and that this interaction alone is sufficient to induces degranulation and IL-6 secretion from mast cells and also to enhance significantly FcεRI-activated secretion of pro-inflammatory mediators.

At least four isoforms of CADM1 are generated through alternative splicing, with resulting changes in glycosylation impacting on cell specific functions and adhesion strength (Fogel et al., 2007; Hagiyama et al., 2011; Moiseeva et al., 2013a). Our protein expression analysis in BMMCs is consistent with other studies on human (Yang et al., 2006; Moiseeva et al., 2012) and rodent mast cells (Ito et al., 2003) showing expression of a single isoform of CADM1 with a m.w. of ∼100 kDa. This is significantly higher than the predicted molecular weight of any of the four *CADM* gene encoded proteins (m.w. 40–45 kDa), but in agreement with the apparent m.w. of fully glycosylated CADM1c isoform. The specific expression of CADM1c in mast cells may be of significance when it comes to controlling adhesion to sensory neurons. It is known that dimer formation is essential for CADM-mediated adhesion, CADM1c is unique among the four common isoforms in forming heterodimers with CADM1d, thereby significantly increasing the strength of adhesion compared to that formed by homodimers (Hagiyama et al., 2011). BMMCs grown in monocultures notably show very little adhesion to each other. This observation suggests that CADM1c does not mediate significant homotypic adhesion and that trans CADM1 binding is isoform dependent (Hagiyama et al., 2011).

CADM1 expression analysis on isolated adult DRGs showed a thick band of protein with an estimated m.w. of ∼75 kDa, the expected molecular weight of isoform d. In other neurons, CADM1d expression is developmentally regulated, concentrated in neurites and linked to formation of synapses (Fogel et al., 2007; Hagiyama et al., 2011). Our immunocytochemistry analysis of DRG indicated that CADM1 was expressed in the soma of all subtypes of sensory neurons and some neurites. When BMMCs were co-cultured with DRG, BMMCs were observed to have attach to neurites where CADM1 was concentrated. In contrast to our results, a previous study investigating mast cell-sensory neuron interactions failed to identify CADM1 expression in DRG and concluded that nectin3 mediated adhesion with mast cells (Hagiyama et al., 2011; Furuno et al., 2012). Differences between the two studies may arise from the age of mice used to isolate DRG and developmental regulation of CADM1 expression. In agreement with our protein expression analysis in adult DRG, single cell RNA sequence analysis of DRG neurons also indicates expression of CADM1 in all subtypes of sensory neurons and interestingly, also expression of CADM2 in nociceptors (Usoskin et al., 2015). CADM1 and CADM2 protein expression throughout development has been reported in chick DRG and interestingly, in the same report confirmed in mouse (Frei et al., 2014). CADM2 also has a mw ∼76 kDa, and forms strong and specific cis-heterophilic interactions with CADM1 (Frei et al., 2014), and could therefore also be contributing to, or regulating, the formation of trans-heterophilic interactions mediating the adhesion of mast cells to sensory neurites observed in our study.

Blocking CADM1 before co-culture reduced the percentage of BMMCs adhered to sensory neurons. Knockdown CADM1 expression from BMMCs also significantly reduced their adhesion to neurons. Despite the effectiveness of the ShRNA in ablating expression of CADM1 in BMMCs, the reduction in adhesion achieved was less than that obtained with the blocking peptide. This difference could arise from non-specific inhibition of other structurally-related immunoglobulin superfamily adhesion receptors by the peptide, such as ICAM-1 (Inamura et al., 1998). Another possibility is that CADM1 knockdown leads to compensatory up-regulation of other adhesion molecules such as integrins which are also widely expressed in mast cells and can regulate their signaling (Sperr et al., 1992; Lorentz et al., 2002; Moiseeva et al., 2014). Nonetheless, taken together the results of both types of interference experiments provide strong evidence that CADM1 is primarily responsible for mast cell adhesion to sensory neurons.

Addition of BMMCs to DRG culture for 1 day did not affect either total neurite length or neurite complexity. Although it is reported that activated mast cells produce NGF and TNFα, which enhance neuronal outgrowth and plasticity (Leon et al., 1994; Kakurai et al., 2006), in our co-culture system, the former, at least was not apparent. Key methodological differences that could account for the lack of morphological changes could be the use of adult versus embryonic DRG (Leon et al., 1994; Frei et al., 2014). Embryonic DRG cultures are dependent on NGF for their survival (Melli and Höke, 2009), while adult DRG cultures are not, despite its receptor expression. Indeed, the role of NGF in adult sensory neurons shifts away from the neurotrophic effect to a pro-inflammatory effect that regulates neuronal function and plasticity (Sofroniew et al., 2001). Our functional studies, as discussed below, support the notion that in adult sensory neurons, the role of CADM1 adhesion is shifted from one supporting axonal pathfinding and formation of neural circuits to one regulating neuroimmune crosstalk.

Functional experiments performed on mono-cultures and co-cultures showed for the first time that CADM1-mediated adhesion between mast cells and sensory neurons specifically and selectively induced degranulation and IL-6 secretion from mast cells and moreover significantly enhanced antigen-activated mast cell responses. While adhesion between the two cell types was very rapid and established within 2 h of contact, the enhancement of mast cell secretory functions was relatively delayed, becoming significant after 6 h of co-culture and continuing to increase over the ensuing 24 h, indicating that activation of CADM1-dependent intracellular signaling pathways are necessary. The observation that mast cell degranulation may be modulated by adhesion to other cells, even in the absence of external stimulation, has been reported previously. Co-culture human lung mast cells with airway smooth muscle show increased constitutive histamine release (Hollins et al., 2008) which starts after 16 h of co-culture (Lewis et al., 2016). Activated T cells enhance constitutive histamine release from BMMC also when co-cultured for 16 h (Inamura et al., 1998). Co-culture of LAD2 mast cells with tumor cells, like pancreatic ductal adenocarcinoma for 24 h also show enhanced constitutive tryptase release (Ma et al., 2013). Here we found that co-culture with HEK cells, which also express CADM1, like sensory neurons, while promoting efficient adhesion, did not however, result in enhanced mast cell secretion implying that additional cell specific interactions facilitated by CADM1-adhesion contribute to the activation and sensitization of mast cells.

Sensory neurons are a potential source of potent mast cell activators, including substance P (Karimi et al., 2000; Okabe et al., 2006), CGRP (Forsythe et al., 2000; Rychter et al., 2011) and ATP (Wareham and Seward, 2016) which may exist as co-transmitters. Exposure to inflammatory mediators drives increased CGRP synthesis and dense core granule biogenesis in peptidergic sensory neurons (Russell et al., 2014). In our co-culture experiments, activation of sensory neurons with capsaicin notably markedly enhanced mast cell degranulation, consistent with the establishment of chemical communication between the cells. Our preliminary experiments with cultures of sensory neurons from trigeminal ganglia, which are enriched in peptidergic neurons, revealed further that co-culture with mast cells was sufficient to significantly increase CGRP secretion (Supplementary Figure 4). It would therefore seem likely that the gradual increase in mast cell degranulation we observed over time in our co-cultures reflect the development of an increasing number of functional neuro-immune synapse like structures between mast cells and sensory neurites, potentiating chemical communication between the two cell types which *in vivo* would translate into pain hypersensitivity (Gupta and Harvima, 2018). The ability of CADM1 to promote pre- and post-synaptic specializations and synaptogenesis in the CNS is well established (Frei and Stoeckli, 2017). Crucial to these synapse promoting functions of CADM proteins, are their highly conserved cytosolic domains which recruit scaffold proteins and effector molecules and increased actin polymerization (Cheadle and Biederer, 2012), which is needed for the capture of dense core vesicles (Porat-Shliom et al., 2013; Bharat et al., 2017). Interestingly, CADM1 driven formation of synapses may be activity driven, and the distribution of CADM1 into membrane nanodomains may physically define the contact edges of synapses (Robbins et al., 2010; Perez de Arce et al., 2015; Ribic et al., 2019). We would therefore propose a model in which the formation of trans-heterotypic CADM1 adhesions between mast cells and sensory neurites leads to the formation and stabilization of neuro-immune synapses enriched in peptidergic vesicles, increasing cross-talk between the two cell types and inducing mast cell activation and secretion. Further studies with super-resolution microscopy will be needed to test this model.

In addition to inducing mast cell degranulation and IL-6 secretion, co-culture with sensory neurons and CADM1-dependent adhesion was also found to enhance antigen-induced responses. Activation of mast cells by antigen is mediated by the crosslinking of FcεRI-receptors which triggers a series of tyrosine kinase regulated phosphorylation events, orchestrated through the engagement of multiple adaptor and scaffold proteins, culminating in the activation of signaling that triggers degranulation, lipid mediator synthesis, and transcription of cytokines and chemokines. In the context of the enhanced FcεRI responses observed in our co-cultures, we found that secretion of TNFα was not potentiated. Transcription of TNFα is driven by NFAT and sustained calcium signaling (Klein et al., 2006; Falvo et al., 2010) whereas IL-6 transcription is driven by NF kappaB signaling, therefore the observed enhancement of only one of the two tested cytokines indicates that CADM1-mediated adhesion of mast cells to sensory neurites augments a selective part of the FcεRI signaling. Precedence for selective potentiation of selective cytokines, and excluding TNFα, has been reported previously and shown to be mediated through synergistic activation of specific kinases regulating the activity of transcription factors. Receptor-mediated inhibition of signaling could also be involved in fine tuning the impact of sensory neuron adhesion on mast cell allergen responses. Activation of GPCRs coupled to pertussis toxin sensitive G*i* proteins, lead to synergistic enhancement of degranulation, IL-6 and TNFα secretion, (Kuehn et al., 2008), which is distinct to what we have observed in co-culture. We observed that activation of mast cells with compound 48/80 was not enhanced by co-culture with sensory neurons. Since compound 48/80 function in mast cells is mediated through activation of MrgprB2 and G*i* proteins, a receptor also targeted by substance P (Subramanian et al., 2016), this suggests however, that the enhancement in antigen-evoked responses observed upon adhesion to sensory neurites does not represent a G*i* PCR-mediated amplification system (Gilfillan et al., 2009). CADM1-mediated interactions with the actin cytoskeleton through its cytosolic domains may also prime FcεRIs in a manner similar to that recently described for integrins in mast cells (Shelby et al., 2016; Wakefield et al., 2017; Halova et al., 2018). While further in-depth molecular studies of FcεRI distribution and signaling cascades are needed to understand how CADM1 adhesion potentiates responses to allergens, our data shows unequivocally that CADM1-mediated adhesion to sensory neurons enhances mast cell derived IL-6 secretion and degranulation and would therefore potentiate mast cell regulated inflammatory responses in atopic individuals. *In vivo*, mast cells of IBS patients secrete greater amounts of the IL-6 (Liebregts et al., 2007) in response to neuronal hyperexcitability (O’Malley et al., 2011). Moreover, IL-6 has been found to induce mechanical nociceptive plasticity (Melemedjian et al., 2010; Hughes et al., 2013) that evokes allodynia (Oka et al., 1995; Dina et al., 2008). Blocking IL-6 receptors in neurons reduces inflammation in an antigen-induced arthritis model (Ebbinghaus et al., 2015) and anti-IL-6 receptor antibodies show promising therapeutic potential in controlling pain in rheumatoid arthritis (Nishimoto et al., 2009). Therefore, there is good evidence already that pain-related disorders may involve an increase in IL-6 levels, such as that reproduced in our co-culture system. In conclusion, we show that CADM1 is necessary to drive mast cells-sensory neuron adhesion and contribute to the development of a microenvironment in which neurons enhance mast cell responsiveness to antigen. This interaction could explain why the incidence of painful neuroinflammatory disorders such as IBS are increased in atopic patients.

## Data Availability

The datasets generated for this study are available on request to the corresponding author.

## Ethics Statement

Healthy, 8–12 week-old male C57BL wild-type adult mice were used in this study. All animals were maintained on a 12-h light/dark cycle in a temperature-controlled environment and given food *ad libitum*. All animal procedures were conducted under the Animal (Scientific Procedures) Act 1986, and approved by the UK Home Office.

## Author Contributions

ES, ZD, and RM designed the experiments. ES and RM prepared the manuscript. RM and JM did the experiments.

## Conflict of Interest Statement

The authors declare that the research was conducted in the absence of any commercial or financial relationships that could be construed as a potential conflict of interest.

## Footnotes

1 www.graphpad.com
