# Supplementary material for: Secretion of Mast Cell Inflammatory Mediators Is Enhanced by CADM1-Dependent Adhesion to Sensory Neurons
Source: Front Cell Neurosci. 2019 Jun 18;13:262. doi: 10.3389/fncel.2019.00262 (PMC6591473; doi:10.3389/fncel.2019.00262)
Supplement: Supplementary file 1 [file Presentation_1.pdf]

Table 1 Antibodies Used for immunocytochemistry and Flow cytometry

Primary Antibodies	Dilution	Supplier	Secondary Antibodies	Dilution	Supplier
Rabbit anti-periphrin	1:1000	Sigma P5117	Alexa Fluor® 488 donkey anti-rabbit	1:1000	Invitrogen A-21206
Rabbit Anti-CADM1 Polyclonal IgG (H-300)	1:300	Santa Cruz sc-33198 lot F0407			
Mouse anti-CGRP	1:100	Abcam Ab81887	Alexa Fluor® 594 donkey anti-mouse	1:1000	Invitrogen A-21203
Mouse anti-B III Tubulin monoclonal IgG	1:1000	R&D MAB1195 clone TuJ-1 lot HGQ0113121			
Goat anti FcεRIγ (A-18) polyclonal IgG	1:300	Santa Cruz (sc-33496 lot: I2811)	Alexa Fluor® 647 chicken anti-goat IgG	1:500	Invitrogen A21449 lot 1700331
Guinea pig anti-SubP	1:100	Abcam ab10353	FITC-donkey anti-guinea pig	1:100	Millipore
Alexa Fluor@ 488 anti-mouse c-Kit	1:100	Biolegend (6861)			
APC-anti-mouse c-Kit (CD117)	1:100	eBioscience (17-1171)			
PE-anti-mouse FceRI	1:100	eBioscience (12-5898)			

Supplementary Figure 1

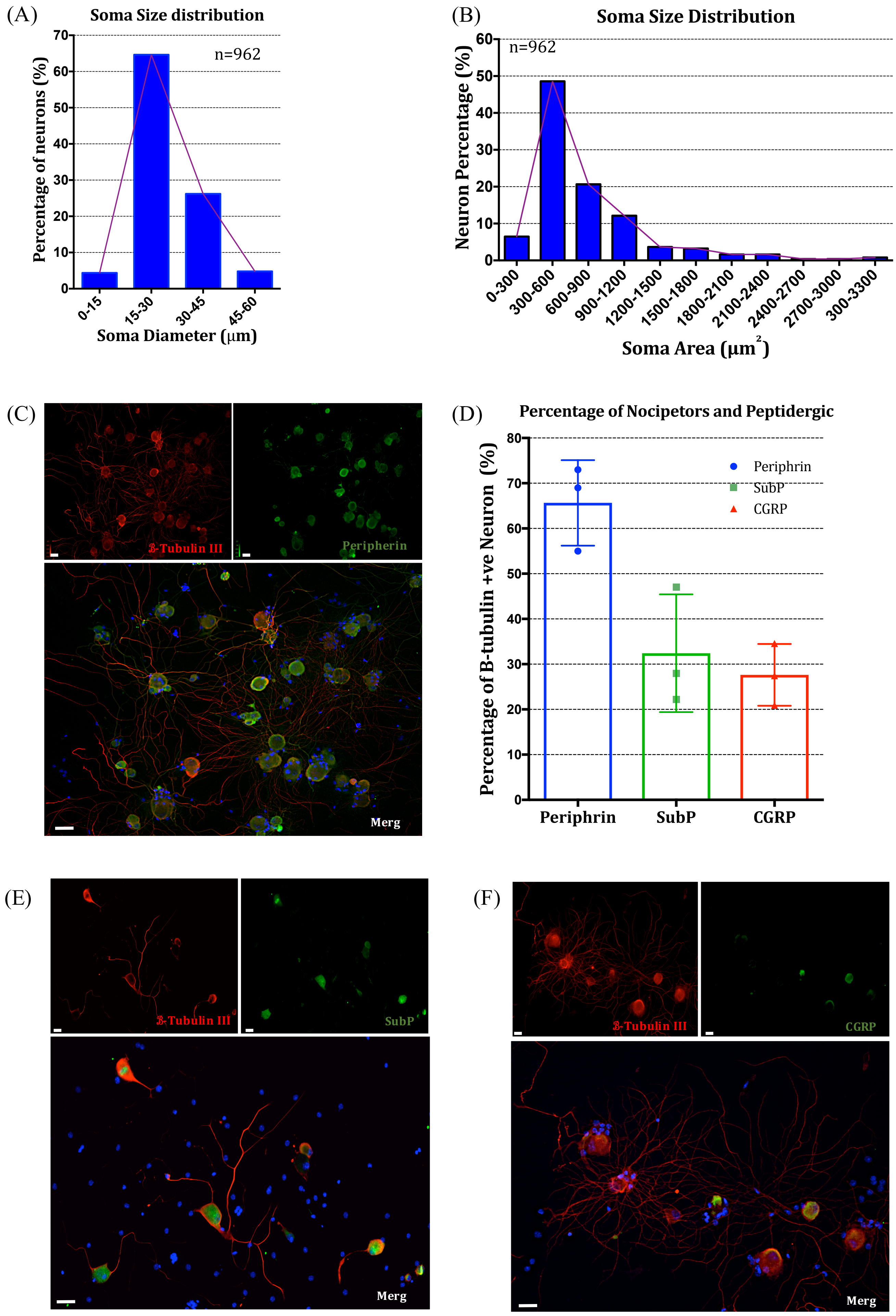

Supplementary Figure 1**. Characterization of neuronal subtypes present in DRG used for co-culture experiments with BMMCs**. Analysis of β-III tubulin-positive neuron soma size distribution on soma diameter (A) and area (B) show enrichment for small nociceptor like cells (<30 μm in diameter). (C) Immunocytochemistry showing distribution of β-III tubulin positive neurons (red) and peripherin positive (green) labelled nociceptors. DAPI staining of nuclei (blue) show presence of non-neuronal cells in the DRG cultures. (D) Percentage of nociceptors positive for peipherin, calcitonin gene-related peptide (CGRP) and substance P (SubP) expressed as a percentage of β-III tubulin immunoreactive neurons (N=3). (E) Immunocytochemistry showing distribution of β-III tubulin-positive neurons (red) and substance P- positive (green) labelled nociceptors, and (F) distribution of β-III tubulin-positive neurons (red) and CGRP- positive (green) nociceptors. CGRP. Scale bars 25μm Small neurons (diameter: < 30 μm and area: <600 μm^2) are considered to be nociceptive while medium (diameter: 30-40 μm and area: 600-1200 μm^2) and large neurons (diameter: > 40 μm and area: >1200 μm^2) are mechanoreceptive (Scroggs and Fox, 1992). More than 60% of neurons in our DRG cultures appear to be small-sized nociceptive neurons. Double staining experiments for peripherin, a neurofilament protein expressed selectively in nociceptive neurons (Goldstein et al., 1991), confirmed that 66 ± 5 % of β III Tubulin-immunoreactive neurons were also positive for peripherin confirming enrichment of the cultures for nociceptors. Quantitative analysis of neurons dual stained with either anti-substance P or anti-CGRP and β III tubulin showed that 32 ± 8 % and 28 ± 4 %of neurons were positive for substance P and CGRP, respectively

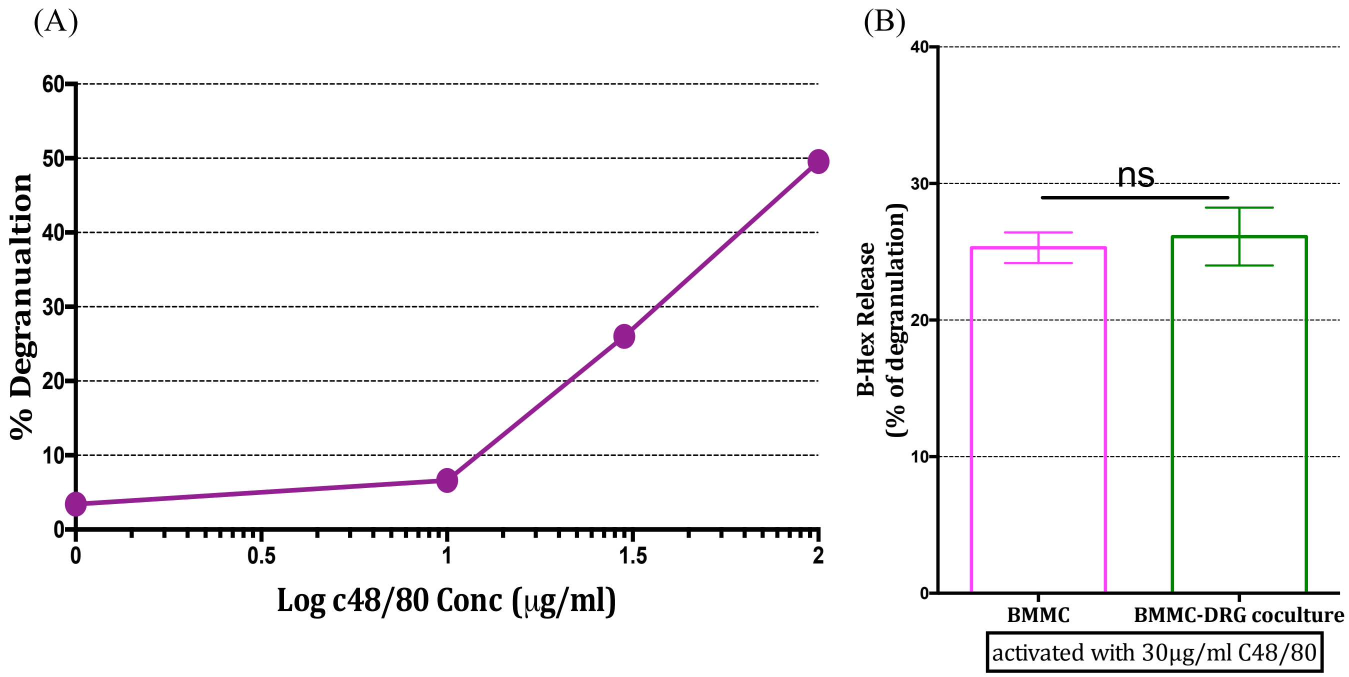

Supplementary Figure 2-**BMMC degranulation induced by compound 48/80 is not potentiated by co-culture with DRG.**
(A) shows concentration response curve for degranulation of BMMCs stimulated for 30 minutes with compound 48/80. The half-maximal concentration (30 μg/ ml) was then used to evaluate the effect of co-culturing BMMCs with DRG neurons on degranulation. (B) Degranulation measured in response to 30 minutes stimulation with compound 48/80 from BMMCs cultured alone or with DRG for 24 hours. Data shown are the mean ± sem for N=3 cultures, each performed in duplicate. Data were analyzed using two-tailed paired t-test. No significant difference in degranulation by compound 48/80 was found between the mono-cultures and co-cultures.

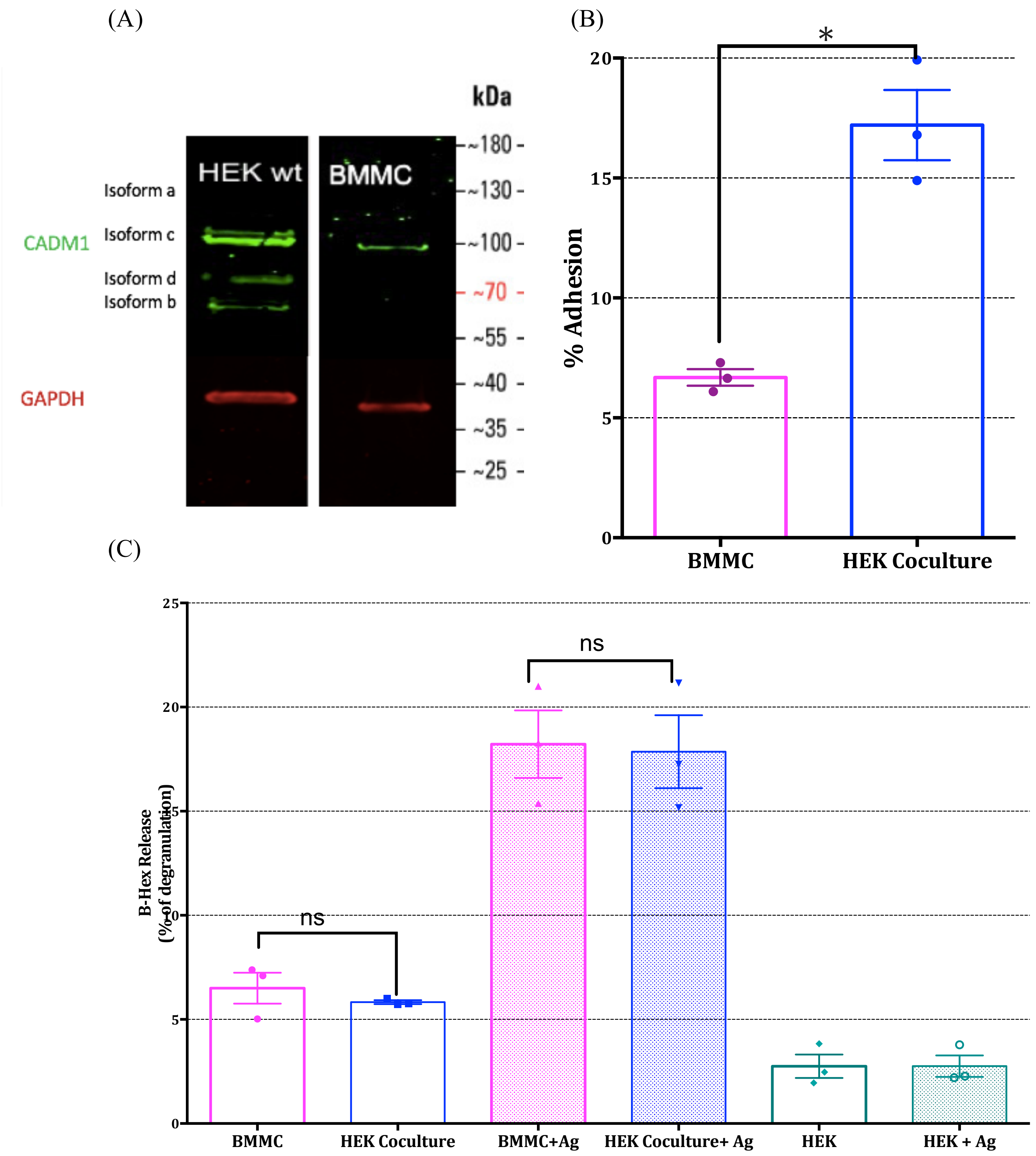

Supplementary Figure 3 – **Adhesion of BMMCs with HEK cells does impact their degranulation. (A) Immunoblot of lysates prepared from HEK and BMMC cultures**. The blots were probed with anti-CADM1 and anti-GAPDH (loading control). The m.w. scale is shown to the right of the blot. Multiple isoforms of CADM1 are expressed in HEK cells, including an isoform of ~70 kDa, similar to that observed in DRG. (B) BMMCs adhesion to HEK cells quantified using the calcein assay and expressed as percentage of adherent BMMC to total BMMC before washing and centrifugation. Data shown as mean ± SEM from N=3. Each done in duplicate. Data were analyzed two-tailed paired *t*-test *$p<0.05$ compared to BMMC culture alone. (C) anti-DNP IGE-sensitized BMMCs were cultured alone or with HEK cells for 24 hours. β-hexosaminidase (β-hex) release was measured in resting condition or after antigen (Ag, DNP) stimulation and expressed as a percentage of total β-hex measured from cells lysed with 0.5% Triton X100. Data shown are mean ± SEM of N=3, each performed in duplicate. ns=non-significant compared to BMMC alone. Data were analyzed using two-tailed paired *t*-test.

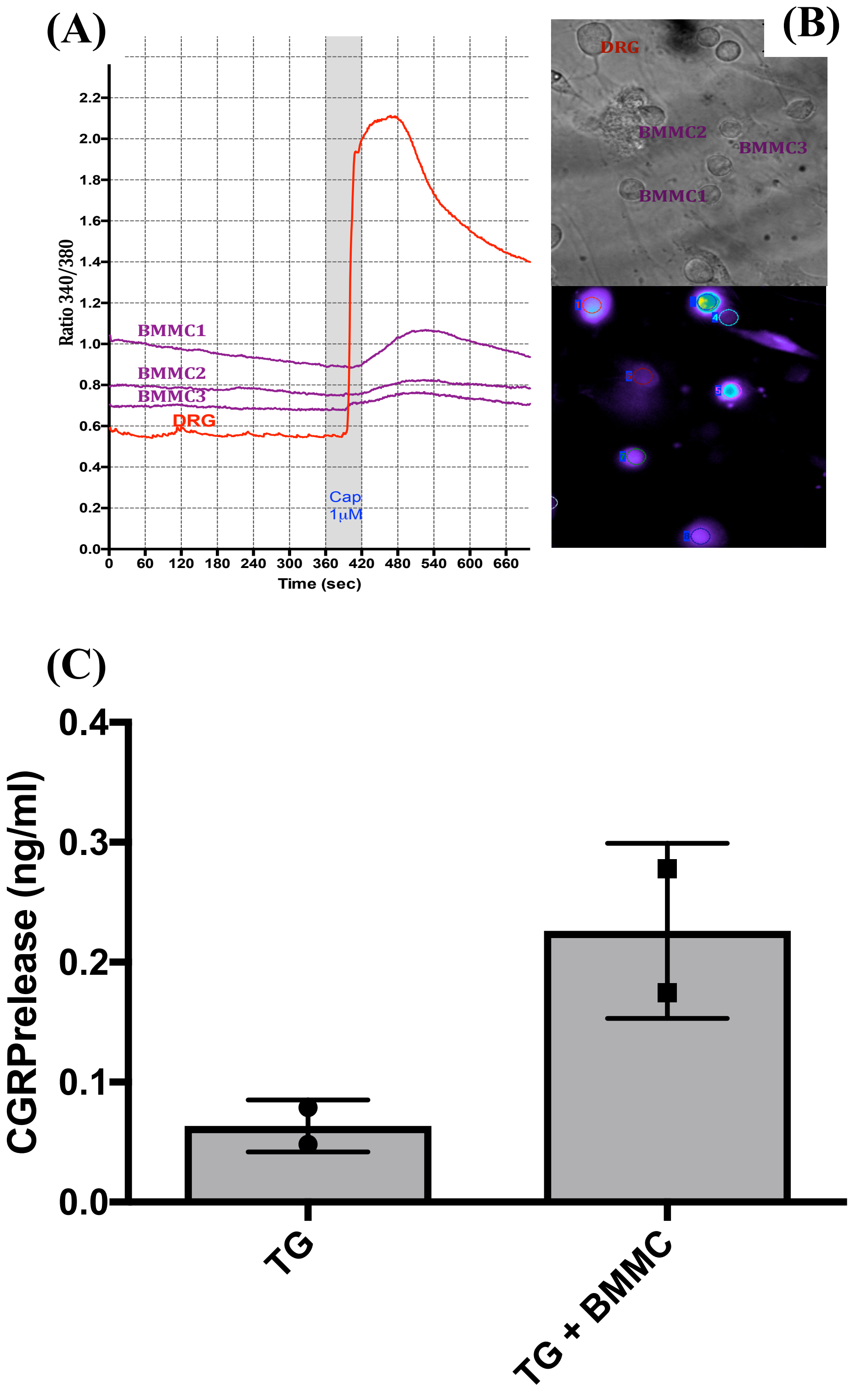

Supplementary Figure 4 - **Evidence of chemical communication between mast cells and sensory neurons in co-culture**.(A) Ratiometric fura-2 imaging of calcium signaling in co-cultures of DRG neurons and mast cells. Cells maintained in co-culture for 24 hours were passively loaded with 1μM Fura-2AM and placed on the stage of an inverted microscope and perfused with physiological saline, washing away any non-adherent mast cells. At the indicated time point, sensory neurons were activated by the addition of capsaicin. Note the large increase in the calcium signal elicited in a representative sensory neuron indicated in (B) is followed by a short delay with an increase in calcium in the indicated mast cells. (B) Transmitted light and fluorescent image of cells imaged in (A). A region of interest was placed over the soma of each of the indicated cells. Cells were excited with alternating light at 340 and 380 nm and emitted light collected at 10Hz and plotted (A). (C) CGRP measured by ELISA in the media recovered from monocultures and co-cultures of dissociated trigeminal ganglion and BMMCs. Data shown is the mean ± sem, n=3.
